# Emerging Evidence in Out-of-Hospital Cardiac Arrest—A Critical Appraisal of the Cardiac Arrest Center

**DOI:** 10.3390/jcm13133973

**Published:** 2024-07-07

**Authors:** Felix Memenga, Christoph Sinning

**Affiliations:** Department of Cardiology, University Heart & Vascular Center Hamburg, 20246 Hamburg, Germany; f.memenga.ext@uke.de

**Keywords:** out-of-hospital cardiac arrest, cardiac arrest center

## Abstract

The morbidity and mortality of out-of-hospital cardiac arrest (OHCA) due to presumed cardiac causes have remained unwaveringly high over the last few decades. Less than 10% of patients survive until hospital discharge. Treatment of OHCA patients has traditionally relied on expert opinions. However, there is growing evidence on managing OHCA patients favorably during the prehospital phase, coronary and intensive care, and even beyond hospital discharge. To improve outcomes in OHCA, experts have proposed the establishment of cardiac arrest centers (CACs) as pivotal elements. CACs are expert facilities that pool resources and staff, provide infrastructure, treatment pathways, and networks to deliver comprehensive and guideline-recommended post-cardiac arrest care, as well as promote research. This review aims to address knowledge gaps in the 2020 consensus on CACs of major European medical associations, considering novel evidence on critical issues in both pre- and in-hospital OHCA management, such as the timing of coronary angiography and the use of extracorporeal cardiopulmonary resuscitation (eCPR). The goal is to harmonize new evidence with the concept of CACs.

## 1. Introduction

Out-of-hospital cardiac arrest (OHCA) is a leading cause of death and an enormous burden on society and healthcare systems. The global average incidence of OHCA is 55 per 100,000 person-years, with an average survival rate of approximately 7% to hospital discharge [1]. While survival rates vary in different regions, with slightly better survival rates in Europe and North America, an improvement in survival rates in OHCA patients who received cardiopulmonary resuscitation (CPR) has been observed over the last 40 years [1,2]. 

Given the poor prognosis, the development of cardiac arrest centers (CACs) has recently been proposed as preferred treatment facilities for all OHCA patients (of presumed cardiac cause), providing comprehensive and high-quality post-resuscitation care [3]. These high-volume facilities (treating preferably at least 40 OHCA patients per year) include an on-site coronary angiography laboratory with 24/7 availability, an emergency department, an intensive care unit, imaging facilities, and a protocol outlining the transfer of selected patients to “OHCA hub hospitals” with additional resources [3]. 

Substantial efforts have been undertaken to encourage bystander CPR in the *pre-hospital* stage of OHCA management, which improves outcomes [4,5,6] and is performed in an increasing number of OHCA cases [3,7]. The same applies to public access defibrillator use in shockable rhythms [8]. Major regional differences in the organization of emergency medical services (EMS), which are organized differently throughout Europe, account for the heterogeneity of pre-clinical OHCA management. Early contact with the receiving CAC within an established protocol is essential to ensuring adequate preparation of staff and facilities. 

OHCA is most often caused by coronary artery disease (CAD) [9], so the *in-hospital* management of OHCA patients after return of spontaneous circulation (ROSC) largely corresponds to the management of acute coronary syndrome (ACS) with or without cardiogenic shock. Treatment decisions evolve around the issues of (a) the timing of invasive coronary angiography (immediate or delayed), dependent on the presence or absence of chest pain before OHCA-onset and ST-segment elevations in the ECG after ROSC, and (b) the extent of percutaneous coronary intervention (PCI, culprit-only or multi-vessel). Patients without ROSC or in cardiogenic shock may be considered candidates for extracorporeal cardiopulmonary resuscitation (eCPR) or mechanical circulatory support (MCS). Comprehensive intensive care management of post-cardiac arrest syndrome with multiple organ failure may further comprise hemodynamic and ventilatory support, renal replacement therapy, and targeted temperature management (TTM). Early neurological prognostication is necessary to identify patients with poor neurologic outcomes due to hypoxic-ischemic cerebral injury to adjust goals of care, initiate organ-preserving intensive care in possible organ donors, and provide adequate rehabilitation care in patients with a presumably good neurological prognosis.

This narrative review aims to present and discuss new evidence on these cornerstones of pre- and in-hospital management of OHCA, and to explore their potential impact on the CAC strategy.

## 2. Methods

In May 2024, we conducted a systematic literature search in the MEDLINE database for clinical trials, meta-analyses, reviews, and systematic reviews using the following search terms: “out-of-hospital cardiac arrest (or OHCA)”, “cardiac arrest”, “sudden cardiac death”, “extracorporeal CPR (or eCPR)”, “post-cardiac arrest care”, “post-resuscitation care”, “cardiac arrest center” and “cardiac arrest registry”. As our search yielded a vast number of publications that might potentially bear implications for future OHCA management, the focus of this narrative review was placed on high-quality evidence (randomized controlled trials, meta-reviews, and systematic reviews) that had been published in the last 4 years. The publications considered relevant will be presented and discussed in the following order, chronologically structured by the sequence of OHCA treatment: pre-hospital care, coronary care, MCS and eCPR, post-cardiac arrest care (with separate sections dedicated to targeted temperature management (TTM) and neuroprognostication and risk assessment tools), rehabilitation and post-discharge care, and management in CACs. We added references to the guideline recommendations of major medical societies, if available.

## 3. Pre-Hospital Care

The beneficial effects of lay CPR (6) and timely use of an AED by members of the public [8] on survival and neurologic prognosis in OHCA are well established. However, despite considerable efforts in public education, awareness campaigns, and training programs, obvious barriers to public intervention in the case of an OHCA remain, such as a simple lack of knowledge or difficulty in locating a public access AED [10]. A smartphone-based trial for dispatching volunteer responders to collect AEDs close to an OHCA site did not lead to an increase in volunteer AED utilization [11]. However, the deployment of trained volunteers as first responders (“super lay-rescuers”) is a promising concept to extend AED use, at least in rural areas, reduce time to defibrillation in shockable rhythms, and thus improve survival [12,13,14]. Recent studies suggest that delivering AEDs to suspected OHCA cases via drones is feasible and may provide a critical time advantage compared to EMS arrival, but the impact of regulatory factors, geographical constraints, weather conditions, and cost-effectiveness need to be further investigated in the future [15,16,17].

## 4. Coronary Care

The benefit of immediate angiography and revascularization of the culprit coronary lesion in *conscious* OHCA patients with an initial shockable rhythm and ST-segment elevation in the 12-lead ECG after ROSC is undisputed [18,19,20,21]. Immediate reperfusion therapy, preferably via primary PCI, is and has been the standard of care in STEMI for more than three decades [18,20,22]. There are, however, no randomized controlled trials in support of immediate angiography in comatose OHCA patients with ST elevation in the post-ROSC ECG as of now, so clinical presentation and findings and suspected neurological prognosis should also be taken into consideration in these patients [19,23].

While an initial shockable rhythm in OHCA is a well-recognized marker of a more favorable prognosis [24], the prognostic impact of rhythm changes has recently been studied in several retrospective registries [25,26,27]. Conversion from an initial non-shockable to a shockable rhythm may be associated with a better neurologic outcome than non-shockable rhythms without changes, but the initial rhythm remains the best prognostic marker [25,26,27].

Determining whether and when to activate the coronary catheter laboratory in the *absence* of ST elevation post-OHCA is challenging. In this setting, an occluded culprit vessel may be present in about 23% of patients [21], but current noninvasive tests still lack the sensitivity to unequivocally identify ongoing coronary ischemia [19]. However, advanced imaging techniques such as the resting LV-GLS (left ventricular global longitudinal strain) in transthoracic echocardiography can predict the presence and severity of CAD [28], and transesophageal echocardiography during resuscitation and after ROSC may provide a more detailed characterization of cardiac activity [29,30]. These techniques may help guide post-OHCA management but have yet to be validated on a larger scale in OHCA.

The role of early vs. delayed coronary angiography and revascularization in OHCA patients without ST-segment elevation after ROSC (but initial shockable rhythm and thus suspected cardiac origin) has recently been studied in three randomized controlled trials: the COACT [31] trial of 2019, the TOMAHAWK trial [32] of 2021, and the EMERGE [33] trial of 2022. All trials were unable to demonstrate a benefit of immediate compared to delayed angiography concerning both short-term [31,34], 180-day [33], and/or 1-year overall mortality and clinical outcomes, particularly neurological impairment [32,33,35]. A recent meta-analysis of 11 trials conducted from 2012 to 2019 regarding this issue (including COACT) similarly concluded that early coronary angiography in OHCA without ST elevation has no impact on 30-day mortality, neurological status, or rate of PCI compared to non-early coronary angiography [36].

Most OHCA patients suffer from multivessel coronary artery disease, and those presenting with STEMI have been shown to have more severe CAD than STEMI patients without OHCA [37]. This raises the question of whether a culprit-only PCI strategy should be preferred to complete revascularization (also of non-culprit coronary artery stenoses). The MULTISTARS-AMI trial found *immediate* multivessel PCI to be non-inferior to staged multivessel PCI (with respect to a composite endpoint including all-cause mortality) in STEMI patients. However, the trial did not investigate an OHCA collective (only 3.8% of the included patients were resuscitated before randomization) [38]. In a collective of 706 patients with cardiogenic shock due to acute myocardial infarction (AMI-CS) with multivessel CAD, more than half of whom (52%) were resuscitated before randomization, a lower incidence of death or severe renal failure at day 30 was achieved by a culprit-only strategy, but 1-year mortality did not differ [39,40]. Despite the scarcity of randomized controlled trials, single-stage multivessel PCI may offer no substantial benefits over the culprit-only strategy in AMI-CS [41].

## 5. MCS and eCPR

Due to the poor prognosis of OHCA and cardiogenic shock, attempts at re-establishing circulation have recently taken on prominence. Besides limited evidence from randomized controlled trials, mechanical circulatory support (MCS) in cardiogenic shock, mostly using venoarterial extracorporeal membrane oxygenation (VA-ECMO), a percutaneous transvalvular microaxial flow pump (Impella, Abiomed, Danvers, MA, USA), or both (as the “ECMELLA” concept of VA-ECMO therapy with Impella-based LV unloading), has substantially increased in the last decade [42,43,44]. While VA-ECMO therapy is accessible in most European countries, there are major spatial disparities as well as persisting uncertainties regarding optimal patient selection, timing, location, and method of implantation [43,45].

In the recent randomized controlled ECLS-SHOCK trial and a subsequent individual patient-based meta-analysis of 4 randomized clinical trials, early *routine* use of VA-ECMO in AMI-CS did not improve all-cause mortality at day 30 compared to medical therapy [46,47]. Instead, ECMO therapy caused more bleeding and vascular events [46,47]. ECLS-SHOCK included a higher proportion of resuscitated patients (78% in either study group) than previous trials [39,43,46]. The data, however, suggest that the volume of VA-ECMO procedures may impact outcomes [48], underlining the CAC strategy. Early active LV unloading may also be crucial [49] and is currently being studied in ongoing randomized trials [50,51]. In STEMI with cardiogenic shock, a recent study investigating the routine use of a microaxial flow pump found a survival benefit after 6 months, but OHCA patients were excluded from the study [52].

In refractory cardiac arrest, bridging with VA-ECMO to maintain organ perfusion may enable crucial diagnostic testing and therapeutic interventions to successfully treat the underlying etiology. This form of salvage therapy has become known as extracorporeal CPR (eCPR) and should ideally be initiated within 60 min of OHCA onset [53]. While observational data indicated a promising survival benefit [54,55], there is limited and conflicting evidence from randomized controlled trials concerning the benefits of eCPR in OHCA. A 2023 randomized controlled trial in 160 patients with refractory OHCA failed to demonstrate a benefit of eCPR over conventional CPR concerning survival with favorable neurologic outcomes at day 30 [56]. The Prague-OHCA randomized trial came to a similar conclusion (at day 180), comparing eCPR in combination with further interventions such as intra-arrest transport and immediate invasive assessment to regular advanced cardiac life support (ACLS) [57]. The 2020 ARREST trial comparing eCPR to standard ACLS treatment in OHCA patients with refractory ventricular fibrillation showed a significant improvement in survival to hospital discharge in the eCPR group and was stopped prematurely after the inclusion of 30 patients (15 in each study group).

Given the conflicting evidence, a recent meta-analysis concludes that data encourages eCPR provision in carefully selected patients, preferably in well-organized high-volume centers with highly trained and coordinated staff, but the benefits of eCPR in in-hospital cardiac arrest (IHCA) may exceed those in OHCA [42].

## 6. Post-Cardiac Arrest Care

Following cardiac arrest, post-resuscitation syndrome with multi-organ failure requires complex, evidence-based, and systematic intensive care to improve survival, promote recovery, and prevent secondary brain injury. Multi-disciplinary management at the Cardiac Intensive Care Unit comprises hemodynamic and ventilatory management, neurologic support including targeted temperature management (TTM) and neuroprognostication (both with more robust evidence, which will be addressed separately in the following sections), and other supportive measures [58]. Despite a lack of high-quality studies and randomized controlled clinical trials, the 2021 post-resuscitation care guidelines of the European Resuscitation Council (ERC) and the European Society of Intensive Care Medicine (ESICM) [59] and the 2024 Scientific Statement from the American Heart Association (AHA) and Neurocritical Care Society (NCS) [60] outline standards of care and best practice based on the available evidence on the critical care of post-cardiac arrest patients. However, for the management of patients *after eCPR*, only limited data and no established guidelines exist as of today [42].

## 7. Targeted Temperature Management (TTM)

Mitigating hypoxic brain injury through induced mild hypothermia in OHCA patients has been shown to improve survival and neurologic outcomes [61,62]. This was first demonstrated in 2002 [62] and subsequently incorporated into international guidelines for post-cardiac arrest care, e.g., the 2010 AHA Guidelines for Cardiopulmonary Resuscitation and Emergency Cardiovascular Care. These guidelines recommended a target temperature of 32–34 °C for 12 to 24 h after ROSC in unconscious OHCA patients with shockable rhythms [63]. However, in the last two decades, several high-quality clinical trials, including the two landmark trials TTM of 2013 [64] and TTM-2 of 2021 [65], have failed to confirm the benefit of TTM at this range, as compared to normothermia [66,67]. In the wake of newly emerging evidence, the AHA guideline update of 2015 and the ERC-ESICM guidelines of 2021 in the meantime recommended TTM at a constant temperature between 32 and 36 °C for at least 24 h (and avoidance of fever for at least 72 h) in comatose OHCA (or IHCA) patients after ROSC [59,68] in the meantime, but recently adjusted these recommendations. A 2023 scientific statement from the AHA concluded that in comatose OHCA patients with similar clinical characteristics to the TTM-2 study population, controlling patient temperature to <37.5 °C was a “reasonable and evidence-based approach” [69], while the ERC-ESICM currently recommends “continuous monitoring of core temperature and actively preventing fever (defined as a temperature > 37.7 °C)” for at least 72 h after ROSC [70].

## 8. Neuroprognostication and Risk Assessment Tools

Accurate neuroprognostication in comatose OHCA survivors is fundamentally important, particularly to identify those with a poor neurological outcome. In these patients, it provides guidance on decision-making, especially in terms of withdrawal of life-sustaining therapy (WLST), but also in terms of resource allocation and economics. According to the Neurocritical Care Society Guidelines of 2023 [71], multimodal neuroprognostication should be carried out 72 h after ROSC and rewarming after TTM. The bilateral absence of pupillary light response and N20 response on somatosensory evoked potential testing that reliably predicted a poor neurological outcome [71].

As neuroprognostication at this delayed time point may only be relevant for a minority of OHCA survivors [72], several cardiac-arrest-specific scoring systems for risk stratification at hospital admission have recently been developed: the well-validated CAHP (Cardiac Arrest Hospital Prognosis) [73] and OHCA score [74], and the less complex, summation-only TTM [75] and MIRACLE_2_ score [76,77]. These scores are based on different combinations of factors associated with unfavorable neurologic outcomes after ROSC following OHCA, such as age, non-shockable rhythm, low-flow and no-flow time, arrest location, epinephrine use, eye reflexes or pH, and serum lactate or creatinine levels upon admission. While all scoring systems proved accurate and reliable risk predictors in a recent analysis [78], the simplicity of the MIRACLE_2_-score may facilitate its widespread implementation in clinical practice and use within trials. However, limitations of these scoring systems must be acknowledged, e.g., estimates of no- and low-flow time may often be inaccurate [23], and heart rhythm changes may not sufficiently be considered despite being of prognostic relevance [24]. Relying solely on prognostication based on clinical parameters upon admission may thus surely only insufficiently meet the complexity, heterogeneity, and dynamics of OHCA after ROSC.

## 9. Rehabilitation and Post-Discharge Care

Survivors of OHCA often experience a significant reduction in their health-related quality of life (HRQoL). Besides demographic characteristics such as older age and female sex, anxiety, depression, and impaired neurocognitive function may contribute to this [79]. To address this, the 2021 ERC-ESICM guidelines recommend assessing physical and non-physical impairments to determine rehabilitation needs before discharge. Additionally, cardiac arrest survivors should receive routine follow-up care that includes screening for cognitive or emotional problems and fatigue and provides information and support for patients and their families [59].

## 10. Management in CACs

Cardiac Arrest Centers (CACs) have been proposed as preferred treatment facilities for all patients with OHCA of presumed cardiac etiology, as these high-volume centers provide the resources, infrastructure, staff, and experience to provide high-quality and guideline-recommended coronary care (including eCPR/mechanical circulatory support after careful patient selection) as well as comprehensive post-resuscitation care in OHCA [3].

There is growing evidence, albeit almost exclusively observational (from databases and registries), that OHCA management in a CAC and regionalization of post-cardiac arrest care may be beneficial. Numerous retrospective studies and several large systemic meta-reviews have demonstrated an association between treatment in a CAC and improved survival (at discharge or day 30) and/or better neurological results [80,81,82,83,84,85,86]. In addition, even long-term survival benefits [87] and improvements in functional outcomes [88] have been reported. In contrast to that, the ARREST trial published in 2023, which is the only randomized controlled trial to investigate the impact of CAC treatment to date, could not establish a 30-day mortality benefit for OHCA patients being transferred to a CAC in the absence of ST segment elevations [89]. Table 1 provides an overview of recent evidence on CACs.

Despite the low level of evidence, the International Liaison Committee on Resuscitation (ILCOR) already in 2015 advocated for the establishment of specialist CACs [90] and did not change their recommendation in response to this recent randomized controlled trial, suggesting that “adult patients with nontraumatic OHCA be cared for in CACs rather than non-CACs” in their 2023 international consensus [91]. An important landmark is the broad and interdisciplinary consensus reached on the implementation of CACs in 2020, bringing together international cardiology societies (European Society of Cardiology [ESC]/Association for Acute CardioVascular Care [ACVC], European Heart Rhythm Association [EHRA], European Association of Percutaneous Coronary Interventions [EAPCI]) and international emergency medicine/critical care societies (European Resuscitation Council [ERC], European Society for Emergency Medicine [EUSEM] and European Society of Intensive Care Medicine [ESICM])(3), representing the “best of both worlds”.

In Germany, certification of CACs started in late 2018, initially as a pilot project, with the aim of building a nationwide comprehensive network of CACs to ultimately improve prognosis in OHCA [92]. By the end of 2023, 5 years after the first audit, 114 hospitals in Germany (and neighboring German-speaking countries) will have been certified as CACs. A 2023 retrospective observational study to elucidate the impact of CAC certification in Germany on outcomes in OHCA found CAC accreditation linked to higher rates of favorable neurological outcomes and unchanged overall survival [93,94]. In addition, a survey among EMS staff revealed that a vast majority of first responders may take the CAC accreditation into account in their admission decision [94,95]. Thus, the CAC accreditation model in Germany might serve as a blueprint for similar undertakings in other countries. However, while interdisciplinary CAC criteria in Germany are clearly defined [92,96], the definition of CAC is not universal and may vary across Europe and worldwide. Also, a “bypass” strategy (transporting OHCA patients exclusively to CACs) would need to account for the resulting increase in OHCA numbers.

## 11. Conclusions

As the prognosis for OHCA is poor, prompt and effective treatment is crucial. Despite a growing number of high-quality studies and randomized controlled trials, many aspects of OHCA management, however, still lack robust evidence. This narrative review presents and discusses recent publications on OHCA management, but it is also necessary to contextualize and “translate” emerging evidence to national and regional particularities. Regional differences in the infrastructure and organization of pre-clinical care/EMS, and hospital sectors, as well as the economic limitations of different healthcare systems, can pose significant barriers to providing and maintaining a high quality of care in OHCA.

Clinical registries such as the GCAR (German Cardiac Arrest Registry) [97] play a crucial role in enhancing our understanding of the immediate and prolonged impacts of OHCA, ensuring nationwide quality assurance, and refining procedures for the treatment and ongoing care of OHCA patients.

Given the complexity of the OHCA patient, efforts must be made to ensure management in the setting of CACs, which provide the necessary resources and expertise required for comprehensive treatment according to the best available evidence and guideline recommendations. The CAC approach improves outcomes and promotes education and further research to continuously optimize best practices for the benefit of these most challenging patients, even though more high-quality data from randomized controlled trials is required to further elucidate their impact on OHCA management.

## Figures and Tables

**Table 1:** Recent evidence on CACs.

Authors	Type of Evidence	Total n° of Patients n	N° of Patients Treated in CAC vs. Non-CAC	Primary Endpoints/ Outcome Measures	% of Coronary Angiographies	Key Outcomes Regarding CACs
**Patterson et al., 2023 [89]**	Randomized controlled trial	862	CAC: 431 (50%) vs. Non-CAC: 431 (50%), 411 vs. 412 included in analysis	All-cause mortality at 30 days	CAC: 231 of 412 (56%), non-CAC: 153 of 410 (37%)	No significant difference, *p* = 0.96 (95% CI 0.9–1.11)
**Yeo et al., 2022 [80]**	Systematic review/ meta-analysis	147.943 (36 studies)	n.a.	(a) Survival to hospital discharge or 30 days (b) Survival to hospital discharge or 30 days with favorable neurological outcome	n.a.	(a) CAC favorable, adjusted OR 1.92 (95% CI 1.59–2.32) (b) CAC favorable, adjusted OR 1.85 (95% CI 1.52–2.26)
**Xin Chun Goh et al., 2022 [81]**	Systematic review/ meta-analysis	82.769 (16 studies)	n.a.	(a) Survival to hospital discharge or 30 days (b) Neurological outcomes at hospital discharge or 30 days	n.a.	(a) CAC favorable, adjusted OR 1.28 (95% CI 1.00–1.64) (b) No significant difference, adjusted OR 0.96 (95% CI 0.77–1.20)
**Jung et al., 2022 [84]**	Observational	95.931	CAC: 23.292 (24.3%) vs. Non-CAC: 72.639 (75.7%)	(a) Survival to hospital discharge (b) Good neurologic recovery	n.a.	(a) CAC favorable, adjusted OR 1.70 (1.60–1.80) (b) CAC favorable, adjusted OR 1.75 (1.63–1.89)
**Vopelius-Feldt et al., 2021 [85]**	Observational	10.650 (4.368 after propensity score matching)	24/7 PPCI center: 5.375 (50.5%) vs. Other hospitals: 5.275 (49.5%) High volume center: 5.216 (49.0%) vs. Other hospitals: 5.434 (51.0%)	Survival to hospital discharge	n.a.	24/7 PPCI centers favorable, adjusted OR 1.69 (95% CI 1.28 to 2.23) High volume centers favorable, adjusted OR 1.41 (95% CI 1.14 to 1.75)
**Chien et al., 2020 [83]**	Observational	6.655 (5.156 after propensity score matching)	CAC: 4.039 (60.7%) vs. Non-CAC: 2.616 (39.3%)	(a) (Association of transport time with) survival to hospital discharge (b) (Association of transport time with) good neurological outcome at discharge	n.a.	(a) CAC favorable *in shockable rhythms*, adjusted OR 2.20 (95% CI 1.29–3.75) for transport time < 8 min and adjusted OR 1.92 (95% CI 1.25–2.94) for transport time ≥ 8 min (b) CAC favorable *in shockable rhythms*, adjusted OR 2.7 (95% CI 1.4–5.22) for transport time < 8 min and adjusted OR 1.92 (95% CI 1.25–2.94) for transport time ≥ 8 min
**May et al., 2019 [88]**	Observational	3.855	High-performing centers: 873 vs. Low-performing centers: 1.311	CPC (Cerebral Performance Category) score at hospital discharge	Unconscious catheterization: High-performing centers 451 (53%) vs. Low-performing centers: 411 (32%)	Center specific risk standardized rates for good functional outcome range from 0.47 (0.37–0.58) to 0.2 (0.12–0.26)
**Yeung et al., 2019 [86]**	Systematic review/meta-analysis	Endpoint (a): 46.164 Endpoint (b): 30.080 (17 studies)	(a) CAC: 18.449 vs. Other hospitals: 27.507 (b) CAC: 3.086 vs. Other hospitals: 587	(a) Survival to 30 days with favorable neurological outcome (b) Survival to hospital discharge with favorable neurological outcome	n.a.	(a) No significant difference (OR 2.92, 95% CI 0.68–12.48) (b) CACs favorable (OR 2.22, 95% CI 1.74–2.84)
**Schober et al., 2016 [82]**	Observational	2.238	High-volume center (>100 cases/year): 378 vs. Medium- and low-volume centers: 483	Survival to 30 days with favorable neurological outcome (CPC 1 or 2)	n.a.	High frequency centers favorable (OR 5.2, 95% CI 1.2–21.7, *p* = 0.025)
**Elmer et al., 2016 [87]**	Observational	987	High-volume center: 680 vs. Medium- and low-volume centers: 307	Predictors of long-term survival	High-volume center: 264 of 680 (39%) vs. Medium- and low-volume centers: 115 of 307 (37%)	High-volume center favorable, adjusted HR for treatment in medium- and low-volume centers: 1.58 (95% CI 1.27–1.95), *p* < 0.001
